# Identification and injury to the inferior hypogastric plexus in nerve-sparing radical hysterectomy

**DOI:** 10.1038/s41598-019-49856-w

**Published:** 2019-09-13

**Authors:** Lei Li, Yalan Bi, Leiming Wang, Xinxin Mao, Bernhard Kraemer, Jinghe Lang, Quancai Cui, Ming Wu

**Affiliations:** 1 Department of Obstetrics and Gynecology, Peking Union Medical College Hospital, Peking Union Medical College & Chinese Academy of Medical Science, Beijing, 100730 China; 2 Department of Pathology, Peking Union Medical College Hospital, Peking Union Medical College & Chinese Academy of Medical Science, Beijing, 100730 China; 3 Department of Pathology, Xuanwu Hospital, Capital Medical University, 45# Changchun Street, Beijing, 100053 China; 4 Department of Obstetrics and Gynecology, University of Tuebingen, Calwerstr. 7, Tübingen, 72076 Germany

**Keywords:** Surgical oncology, Cervical cancer

## Abstract

Waterjet dissection of the inferior hypogastric plexus (IHP) resulted in a more rapid return of normal urodynamics than blunt dissection (control group) in patients who received laparoscopic nerve-sparing radical hysterectomy (NSRH) in a randomized controlled study. However, the definite reasons for these results were unknown. This subgroup analysis compared the neural areas and impairment in the IHP uterine branches harvested during NSRH as an alternative to the IHP vesical branches between the waterjet and control groups. This study included samples from 30 eligible patients in each group of the trial NCT03020238. At least one specimen from each side of the IHP uterine branches was resected. The tissues were scanned, images were captured, and the neural component areas were calculated using the image segmentation method. Immunohistochemical staining was used to evaluate neural impairment. The control and waterjet groups had similar areas of whole tissues sent for evaluation. However, the control group had significantly fewer areas (median 272158 versus 200439 μm^2^, p = 0.044) and a lower percentage (median 4.9% versus 3.0%, p = 0.011) of neural tissues. No significant changes in immunohistochemical staining were found between the two groups. For patients with residual urine ≤100 and >100 ml at 14 days after NSRH (42 and 18 patients, respectively), there were significantly different percentages of neural tissues in the resected samples (p < 0.001). Hence, Due to the accurate identification of IHP during NSRH, the waterjet dissection technique achieved better urodynamic results.

## Introduction

Cervical cancer is the fourth most common cause of female cancer-related death worldwide^1^. China also has a high burden of disease and a high prevalence of advanced-stage disease^2^. Radical hysterectomy (RH) is the classical approach for the surgical treatment of early-stage cervical cancer. However, patient quality of life is intensively influenced by RH due to a high rate of postoperative morbidities involving the pelvic autonomic nervous system, including bladder dysfunction, colorectal disorders and sexual dissatisfaction^3^. Nerve-sparing radical hysterectomy (NSRH) was suggested to reduce postoperative bladder function impairment by precise identification of visceral nerve fibers and relevant surgical landmarks, thereby improving the preservation of the neural tissues of the parametrium^4–6^. One of the principle nerve-sparing steps is the preservation of the inferior hypogastric plexus (IHP) during the resection of the cardinal ligament. Dorsal to the parametrial vessels, the plexus at the level of the deep uterine vein^7,8^. Due of the delicate anatomy, damage to the IHP and its vesical branches would cause a waste of effort in other nerve-sparing steps^9^. In a previously reported randomized trial (NCT03020238, *clinicaltrials.gov*), we found that, in laparoscopic NSRH, waterjet dissection of the IHP would result in a rapid return of normal urodynamics without compromising the survival prognosis^10^. However, the potential reasons were unknown.

**Figure 1:**
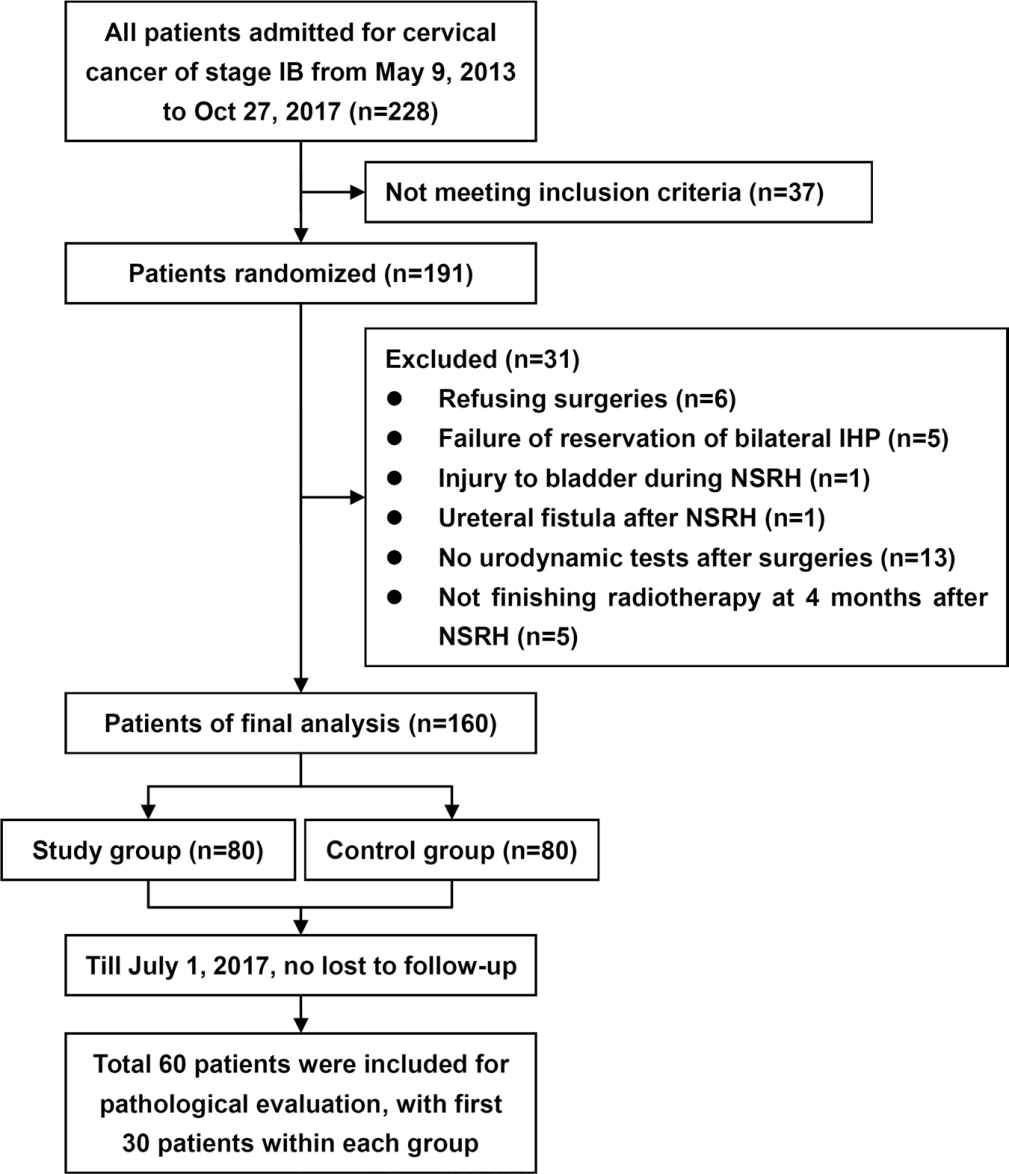
Flow diagram of the study. IHP, inferior hypogastric plexus. NSRH, nerve-sparing radical hysterectomy.

As a subgroup analysis of the trial NCT03020238, we evaluated the neural tissues from the IHP uterine branches harvested during NSRH (Fig. 1). The actual proportion of neural tissues in the resected samples and the degree of impairment in uterine branches were used as an alternative and are representative of the IHP vesical branches. By comparing the neural areas and their proportions among the harvested specimens, we aim to reveal the role of different energy equipments in IHP dissection and the subsequent impact on bladder functions.

## Methods

### Ethical approval

The Institutional Review Board (IRB) from Peking Union Medical College Hospital approved the study (No. S-637 and ZS-1427). The registration numbers were NCT03020238 (PUMCH-OBGYN-2013, registered on January 13, 2017) and NCT03291236 (SOCM-1, registered on September 25, 2017) (*clinicaltrials.gov*). All procedures involving human participants in the study were in accordance with the ethical standards of the IRB from the study center and National Research Committee, and with the 1964 *Declaration of Helsinki* and its later amendments or comparable ethical standards.

### Statement attesting to informed consent for study participation

All patients provided informed consent before treatment, and all the consents were recorded and retained by the researchers in the case reports, which could be copied and published in anonymous way.

### Study design, patient enrollment and sample size

This is a pathological evaluation of specimens from a previously reported randomized controlled study (Fig. 1)^10^. In this study, patients with stage IB cervical cancer (FIGO 2009) accepted type C laparoscopic NSRH. They were randomized into the waterjet and blunt dissection (control) groups according to the different dissection method used to access the IHP. The study group underwent the waterjet method (Erbe Elektromedizin GmbH, Waldhoernlestrasse 17 72072 Tuebingen, Germany). An applicator with a curved tip was adopted to selectively and gently dissect tissue structures, which allowed blood vessels and nerves to remain intact up to a pressure of 35–60 Bar to dissect the middle vesical veins (MVVs), inferior vesical veins (IVVs) and IHP. The control group underwent conventional blunt dissection methods. The inclusion and exclusion criteria were described elsewhere^10^.

Due to the cost of pathological evaluation, samples of the IHP uterine branches were only harvested for additional pathological evaluation in the first 30 eligible patients within each group (Fig. 1). At least one specimen from each side of the IHP uterine branches was resected from included patients.

The primary endpoint was the proportion of neural tissue in the whole sample of cross sections, which is indicated as a percent. The secondary endpoint was the degree of neural tissue impairment, which was illustrated by immunohistochemical (IHC) staining techniques. The urodynamic parameters, residual urine (RU) at 14 days and bladder functions at 4 months after NSRH were evaluated to illustrate the potential relationship between neural changes and bladder functions.

### Surgical treatment and urodynamic tests

**Figure 2:**
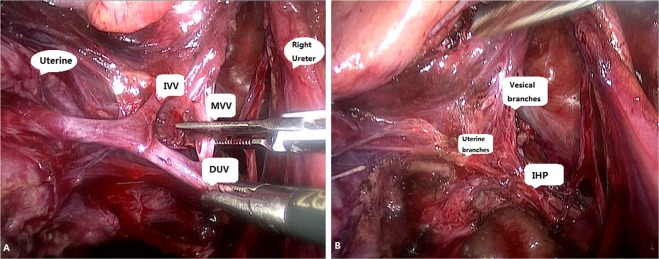
Illustration of the right parametrium. (**A**) The drainage of the inferior vesical vein (IVV) and middle vesical vein (MVV) to the deep uterine vein (DUV), an anatomic landmark of the cardinal ligament, is displayed. (**B**) After removal of the vessels, the inferior hypogastric plexus (IHP) with its vesical and uterine branches were displayed. In our study, the uterine branches of the IHP were resected for pathological evaluation, and the vesical branches were deliberately reserved.

All patients accepted laparoscopic NSRH of type C. All NSRH procedures were performed according to type C1 RH of the Querleu and Morrow (Q-M) classification^11,12^. After truncation of the uterine deep veins and the principle body of the cardinal ligaments, the pelvic splanchnic nerves (PSNs) beneath were sufficiently exposed. Up to this point, the study group and control group were subjected to the same surgical procedures. After clamping and pulling the peduncles of the uterine deep veins, a waterjet was used in the study group to clear up the adipose tissue and distinguish the vessels and nerves, while a suction apparatus with blunt dissection was used to treat the parametrium in the control group. Once the alignment and terminus of the MVVs and IVVs were determined, the MVVs and IVVs were cut off close to the bladder (Fig. 2). After recognition and amputation of the uterine branches of the IHP, the main bodies of the IHP were separated from the parametrium, and the vesical, rectal and vaginal branches of the IHP were preserved. Surgical outcomes, including complications and the application of adjuvant therapy, were consistent with a previous report (Supplement Tables 1 and 2)^10^.

The waterjet dissector (ERBEJET® 2) compatible with the ERBE Workstation (Erbe Elektromedizin GmbH, Waldhoernlestrasse 17 72072 Tuebingen, Germany) with hybrid technology has been described before^10^. An applicator with a curved tip was adopted to dissect tissue structures under different water pressure of 35–60 bar caused by the module selectively, which allowed blood vessels and nerves to remain intact.

Urodynamic tests were performed at 14 days and bladder function tests at 4 months (±2 weeks) after NSRH (Supplement Table 3)^10^. At 14 days after NSRH, for patients with RU > 100 ml, the catheter was kept in place until removal of the catheter was appropriate.

### Pathological evaluations of the IHP uterine branches

For patients included in the study, amputated IHP uterine branches were sent for pathological evaluations according to the conference with pathologists involved in the study (YB, LW and QC). According to the suggestion and practical utilization, the evaluations included (1) neural tissue areas and the proportion among the whole harvested tissues and (2) the degree of impairment of the neural tissues. The actual proportion of neural tissues in the resected samples and their degree of impairment were used as an alternative and are representative of the IHP vesical branches. The detailed methods are described in Supplement 1.

**Figure 3:**
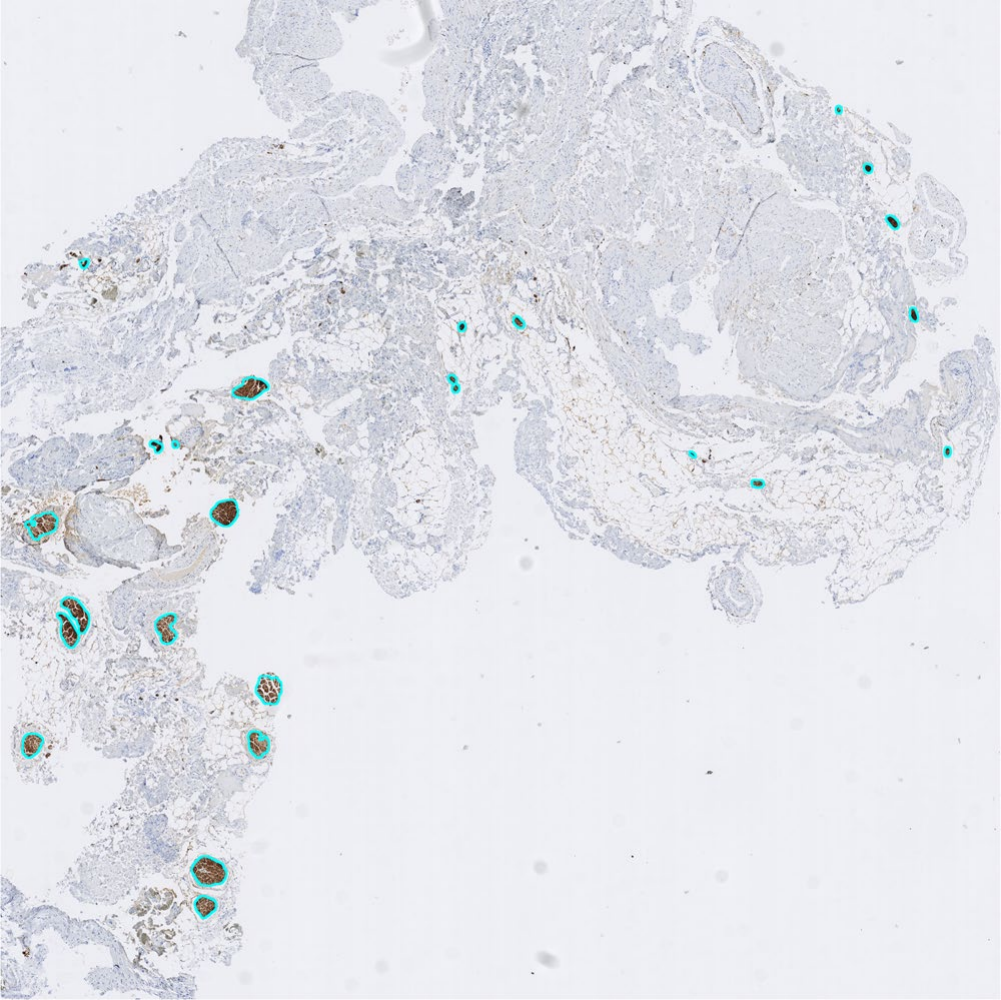
An illustration of quantitative analysis of the neural tissue proportion in a cross section of uterine branches of the inferior hypogastric plexus (×20). The indigo circles denote the neural tissue captured by the image segmentation method. A more detailed description is provided in Supplement 1.

The neural tissues from each side of the IHP were scanned and captured by a scanner, and the areas were calculated using a threshold-based image segmentation method. The total estimated neural tissue area, as well as the total tissue area, was computed for each whole-slide image (Fig. 3).

**Figure 4:**
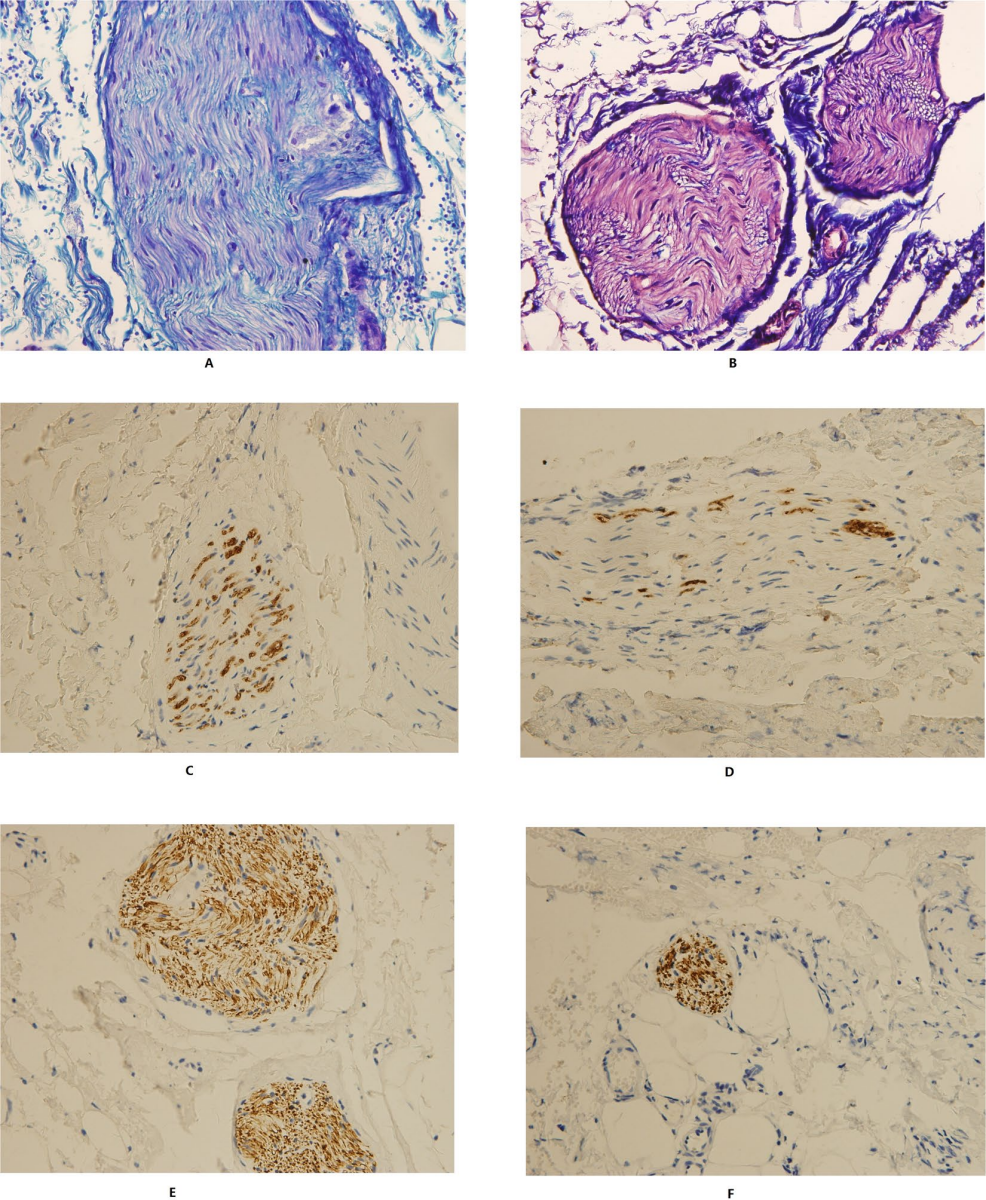
Images of specific staining for nerve tissues. (**A**) Normal myelin in luxol fast blue (LFB) staining. (**B**) Mild myelin decrease in LFB staining. (**C**) Normal expression of myelin basic protein (MBP). (**D**) Mild myelin decrease in MBP expression. (**E**) Normal expression of neurofilament (NF). (**F**) Mild decrease of NF expression.

The samples with the maximum proportion of neural tissues from each side of the IHP were selected for immunohistochemical staining of myelin basic protein (MBP), neurofilament (NF), S100 protein and histochemical staining of luxol fast blue (LFB). MBP and LFB staining were used to show the myelin sheath of peripheral nerves, while NF staining was used to show the axons. Additionally, S100 protein staining illustrated the activities of Schwann cells around the peripheral nerves. An example refer Fig. 4.

### Statistics

Statistic analysis was used for the comparison of clinicopathological characteristics, urodynamic parameters, and neural tissue areas, specifically the percent of total area and the impairment degree of neural tissues. Comparisons of continuous variables were conducted with parametric methods if assumptions of normal distribution were confirmed. Nonnormally distributed variables and categorical data were compared between the two groups using the Mann-Whitney *U* test. Unless otherwise stated, all analyses were performed with a two-sided significance level of 0.05 and were conducted with the use of the software SPSS 22.0 (SPSS, Inc., Chicago, IL, USA).

## Results

### Patients’ clinicopathological characteristics

The general clinicopathological, urodynamic and survival data are listed in Supplement 2. The quantitative analysis and specific staining of IHP uterine branches are listed in Table 1 and Supplement 3. All the baseline epidemiological characteristics of the waterjet and control groups were well balanced, except that patients in the control group were younger (Supplement Table 1). All the surgical and pathological characteristics of the two groups were well balanced, except the control group had a shorter surgical duration and more severe complications within 3 months after NSRH (Supplement Table 2).

**Table 1:** Quantitative analysis and staining of uterine branches of inferior hypogastric plexus.

	Waterjet group (*N* = 60)	Control group (*N* = 60)	*P*
Areas of neural tissue (μm^2^), median (range)	272158 (1774–2115680)	200439 (4285–1353863)	0.044
Left side (n = 30)	308609 (16770–1406458)	191030 (4285–754113)	0.028
Right side (n = 30)	245147 (1774–2115680)	228393 (6879–1353683)	0.525
Areas of whole tissue (μm^2^), median (range)	10262678 (916084–23771968)	8940016 (1523285–16685758)	0.378
Left side (n = 30)	10074523 (1666963–22469672)	8983735 (3034569–16685758)	0.723
Right side (n = 30)	10492605 (916084–23771968)	8864996 (1523285–16337897)	0.383
Percent of neural tissue (%), median (range)	3.3 (0.2–19.4)	2.5 (0.1–13.1)	0.048
Left side (n = 30)	3.7 (0.2–19.4)	2.3 (0.1–6.5)	0.013
Right side (n = 30)	2.9 (0.2–13.7)	2.9 (0.1–13.1)	0.690
Maximum percent (n = 30)	4.9 (0.6–19.4)	3.0 (0.3–13.1)	0.011
Percent categories of neural tissue, n (%)			0.061
<1%	12 (20.3%)	20 (33.3%)	
1% to 10%	39 (66.1%)	38 (63.3%)	
>10%	8 (13.6%)	2 (3.3%)	
Staining of S-100 protein, n (%)			0.110
Normal	52 (86.7%)	45 (75.0%)	
Mild increased	7 (11.7%)	15 (25.0%)	
Moderate increased	1 (1.7%)	0 (0.0%)	
Staining of NF, n (%)			0.782
Normal	46 (76.7%)	43 (71.7%)	
Mild decreased	10 (16.7%)	13 (21.7%)	
Moderate decreased	4 (6.7%)	4 (6.7%)	
Staining of MBP, n (%)			0.847
Normal	30 (50.0%)	30 (50.0%)	
Mild decreased	23 (38.3%)	20 (33.3%)	
Moderate decreased	6 (10.0%)	9 (15.0%)	
Severe decreased	1 (1.7%)	1 (1.7%)	
Staining of LFB, n (%)			0.346
Normal	43 (71.7%)	43 (71.7%)	
Mild decreased	17 (28.3%)	15 (25.0%)	
Moderate decreased	0 (0.0%)	2 (3.3%)	

Every patient had two samples (left and right) for analysis and compare. A more detailed data refer Supplement Table 5. NF, neurofilament staining. MBP, myelin basic protein staining. LFB, luxol fast blue staining.

### Urodynamic outcomes

All the urodynamic parameters before surgeries were also well balanced, but the control group had lower bladder compliance at strong desire to void (Cves at SDV) than the waterjet group. However, at 14 days after NSRH, the control group had significantly fewer patients with RU ≤ 100 ml (83.3% versus 56.7%, p = 0.024) than the waterjet group. At 4 months after NSRH, patients in the control groups had lower Cves at SDV. Self-control comparisons of pre- and postoperative urodynamic parameters suggested that patients in the control group had a decreased maximum flow rate (Qmax) (median 28 [range 11–79] versus 22 [12–60] ml/s, p = 0.049) and Pves at Qmax (median 43 [range 23–73] versus 42 [19–72] cm H_2_O, p = 0.017). The patients in the waterjet group had similar bladder functions before and after NSRH. These results accorded with our previous findings^10^.

### Proportion and impairment of IHP uterine branches

In total, 96 and 118 specimens were harvested from the control and waterjet groups, respectively, with median values of 3 (range 1 to 8) and 3 (range 2 to 6) in each group (p = 0.637). After selecting specimens with the maximum proportion of neural tissues on the left and right parametrium, the control and waterjet groups had similar areas of whole tissues sent for evaluation (Table 1). However, the control group had significantly smaller areas of neural tissues (median 272158 versus 200439 μm^2^, p = 0.044) and a lower proportion of neural tissues (median percent 3.3% versus 2.5%, p = 0.048; median maximum percent of both parametrium 4.9% versus 3.0%, p = 0.011) than those in the waterjet group. There were 6 patients (20.0%) with 8 samples in the waterjet group and 2 patients (6.7%) with 2 samples in the control group who had neural tissues >10% of the resected tissues; there were also 11 patients (36.7%) with 12 samples in the waterjet group and 14 patients (46.7%) with 20 samples in the control group who had neural tissues accounting for <1% of the resected tissues.

No significant changes were observed between the waterjet and control groups in IHC staining S-100 protein, NF, MBP or LBP staining. Generally, few instances of severe damage to the myelin sheath or to the neuronal axon occurred (Table 1). In the waterjet group, different waterjet pressures also exhibited similar destruction on the neural tissue according to the various staining methods.

### Relationship between neural proportions and urodynamic parameters

Patients with RU ≤ 100 ml and >100 ml at 14 days after NSRH (42 and 18 cases, respectively) had significantly different percentages of neural tissue (median maximum percent 4.9% [range 0.9–14.1] and 2.1% [0.3–19.4], p < 0.001). However, self-control comparisons of pre- and postoperative urodynamic parameters in these patients did not reveal significant changes.

For patients with a maximum percentage of neural tissues <10% (52 cases), self-control comparisons of pre- and postoperative urodynamic parameters revealed significantly decreased Pdet at Qmax (14.6 ± 2.0 versus 14.5 ± 2.0 cm H_2_O, p = 0.043). For patients with a maximum percentage >10% (8 cases), self-control comparisons of urodynamic parameters showed similar pre- and postoperative results.

## Discussion

In this study, we dissected and resected the IHP uterine branches during NSRH as an alternative to the IHP bladder branches. The hypothesis of our design is that the resected samples (from uterine branches) could present the whole situation of inferior hypogastric plexus. If more neural tissues were preserved in the resected samples due to less traumatic methods, we could conclude that more tissues could be preserved in the vesical branches, and as a consequence better voiding functions were maintained. A quantitative analysis revealed that waterjet dissection may identify more neural tissues in terms of both area and proportion, which were associated with few patients with RU < 100 ml at 14 days after NSRH. Besides, long-term differences in the urodynamic results were also observed between the waterjet and control groups, which accords with previous report^10^. There were two studies on the various energy equipment in resection/dissection of the parametrium for NSRH, including the cavitron ultrasonic surgical aspirator^13^ and an ultrasonic scalpel combined with a vascular clip^14^, both of which revealed better bladder functions after NSRH. However, these studies were retrospective characteristics, and neither provided histological evidence for better identification of IHP.

In our study, IHC analysis revealed a similar, mild impairment of the IHP in both groups, suggesting that different energy equipment had no substantial differences except for the accuracy of neural tissue identification. Numerous reports have discussed the details about the anatomy and neurological function of IHP^15–18^, but only several studies have been conducted on the IHC analysis of pelvic nerves^19–24^. There were fewer reports about the IHC method for IHP recognition^19–21^, and almost none compared the areas, proportion or impairment of nerve fibers as we did. A study on 15 donated female cadavers revealed that the cardinal ligament did not contain the PSNs, and a well-defined fascial structure existed in the bottom or dorsal margin of the cardinal ligament area. The pelvic plexus was separated from the vascular components of the cardinal ligament^19,20^. Using IHC, Kraima *et al*.^21^ found that the vesical plexus is located in both layers of the vesico-uterine ligament and has a very close relationship with the distal ureter, and the distal ureter should be regarded as a risk zone in which the vesical plexus can be damaged.

Our study suggested that even for physicians who are experienced with RH, definitive identification of the IHP was challenging. Although the percentage of neural tissue in the parametrium is not known, it is surprising that most assumed “neural tissues” resected during the NSRH procedures contained very few true nerve fibers. These findings suggest difficulties in identifying the IHP. In a report studying the proteomic analysis of the pelvic autonomic nerve, both soft tissue and vesical branch of the IHP nerve from five women were collected during surgery, and the existence of nerve fiber was not confirmed in one case (20%)^25^. A report determined the nerve area density in cross sections of resected parametria at various distances from the cervix using a point counting technique. The authors found that modified RH is less radical and spares nerves^22^. However, no oncologic or urodynamic results were compared. There have been a few studies on the intraoperative identification of pelvic nerves in NSRH. Modified leucomethylene blue^26^, electrical stimulation^27,28^, and electromyography^29^ were utilized for this purpose and may help improve bladder function during NSRH. Nevertheless, these studies did not present a rigorously controlled design.

We first utilized an imaging method with artificial intelligence to calculate the neural areas and intensity. The method provided precise quantitative analysis. However, these findings were achieved in postoperative specimens. Future imaging techniques could probably provide more detailed, individualized identification of pelvic nerves. The pelvic autonomic nerves and their related organs can also be reconstructed on the basis of MRI findings to present 3D anatomical information^30^. A study of the pelvises of 3 human female fetuses was performed using computer-assisted anatomic dissection and revealed that no dissection should be performed under the crossing point of the ureter and the uterine artery to avoid injury to the vesical efferences of the IHP^31^.

The most important strength of our study is its prospective, randomized design, which may reduce the selection bias. There are several limitations to our study. First, the general quality of life and sex, gastrointestinal functions were not evaluated in our study, so these outcomes remain debatable. A multicenter retrospective study suggested that laparoscopic total mesometrial resection is associated with improved long-term urinary autonomic functions and worse gastrointestinal autonomic outcomes^32^. Second, due to the cost of pathological testing, not all the surgical patients in the study were included. The limited sample size would probably cause observation and selection bias. Third, the accuracy and adequacy of using uterine branches as an alternative to the IHP vesical branches should be validated in future studies. The uniform nerve-sparing steps are the cornerstone of accurate pathological evaluations. In our study, all the essential procedures were performed by one author (MW), which could probably decrease bias caused by various techniques. Fourth, the reproducibility of the imaging-based calculations should be tested in analogous studies. Last but least, although the waterjet had advantages in urogenital surgeries regarding the preservation of sexual function^33^ and the improvement in patients’ postoperative quality of life^34^, the surgeon must be aware of the limitations and risks of spray dispersion when using surgical debridement devices such as the waterjet^35^, especially in mini-invasive surgeries, which have shown inferior survival outcomes in early stage cervical cancer^36^. Although this is a pathological evaluation from a randomized controlled study, and all the baseline epidemiological and clinicopathological characteristics were well balanced, the impact of nerve-sparing methods on the voiding functions needs more stringent examination considering more risk factors.

## Conclusion

Compared with blunt dissection, waterjet dissection of the IHP resulted in better identification and protection of nerve fibers during NSRH without severe damage to the tissue. Improved urodynamic results were observed in the waterjet group as a consequence of delicate dissection of IHP.

## Supplementary information

Supplement Table 1

Supplemental Table 2

Supplemental Table 3

Supplement 1

Supplement 2

Supplement 3

Supplement 4

## References

[1] Siegel RL (2016). Cancer statistics, 2016. CA Cancer J Clin..

[2] Chen W (2016). Cancer statistics in China, 2015. CA Cancer J Clin..

[3] Hazewinkel MH (2010). Long-term cervical cancer survivors suffer from pelvic floor symptoms: A cross-sectional matched cohort study. Gynecol Oncol..

[4] Xue Z (2016). Comparison of Nerve-Sparing Radical Hysterectomy and Radical Hysterectomy: a Systematic Review and Meta-Analysis. Cell Physiol Biochem..

[5] Kim HS (2015). Conventional versus nerve-sparing radical surgery for cervical cancer: a meta-analysis. J Gynecol Oncol..

[6] Kietpeerakool C (2019). Nerve-sparing radical hysterectomy compared to standard radical hysterectomy for women with early stage cervical cancer (stage Ia2 to IIa). Cochrane Database Syst Rev..

[7] Maneschi F (2014). Urodynamic study of bladder function following nerve sparing radical hysterectomy. J Gynecol Oncol..

[8] Rob L (2010). Nerve-sparing and individually tailored surgery for cervical cancer. Lancet Oncol..

[9] Fujii S (2008). Anatomic identification of nerve-sparing radical hysterectomy: a step-by-step procedure. Gynecol Oncol..

[10] Li L (2019). The Urodynamics and Survival Outcomes of Different Methods of Dissecting the Inferior Hypogastric Plexus in Laparoscopic Nerve-Sparing Radical Hysterectomy of Type C: A Randomized Controlled Study. Ann Surg Oncol..

[11] Cibula D (2011). New classification system of radical hysterectomy: emphasis on a three-dimensional anatomic template for parametrial resection. Gynecol Oncol..

[12] Querleu D (2008). Classification of radical hysterectomy. Lancet Oncol..

[13] Hao, M. *et al*. Cavitron Ultrasonic Surgical Aspirator in Laparoscopic Nerve-Sparing Radical Hysterectomy: A Pilot Study. *Int J Gynecol Cancer* (2016). 10.1097/IGC.0000000000000628 PMC4767107 26807637

[14] Zhao D (2018). Limited energy parametrial resection/dissection during modified laparoscopic nerve-sparing radical hysterectomy. Chin J Cancer Res..

[15] Kessler TM (2007). Increased proximal urethral sensory threshold after radical pelvic surgery in women. Neurourol Urodyn..

[16] Li H (2015). Anatomical basis of female pelvic cavity for nerve sparing radical hysterectomy. Surg Radiol Anat..

[17] Park NY (2010). Laparoscopic pelvic anatomy of nerve-sparing radical hysterectomy. Clin Anat..

[18] Niikura H (2007). Surgical anatomy of intrapelvic fasciae and vesico-uterine ligament in nerve-sparing radical hysterectomy with fresh cadaver dissections. Tohoku J Exp Med..

[19] Kato T (2002). Does the cardinal ligament of the uterus contain a nerve that should be preserved in radical hysterectomy?. Anat Sci Int..

[20] Kato T (2003). A new perspective on nerve-sparing radical hysterectomy: nerve topography and over-preservation of the cardinal ligament. Jpn J Clin Oncol..

[21] Kraima AC (2016). Careful Dissection of the Distal Ureter Is Highly Important in Nerve-sparing Radical Pelvic Surgery: A 3D Reconstruction and Immunohistochemical Characterization of the Vesical Plexus. Int J Gynecol Cancer..

[22] Barbic M (2012). Comparison of nerve content in removed parametrial tissue after classic radical hysterectomy and nerve-sparing radical hysterectomy–histologic evaluation. Eur J Gynaecol Oncol..

[23] Chen C (2014). Neurovascular quantitative study of the uterosacral ligament related to nerve-sparing radical hysterectomy. Eur J Obstet Gynecol Reprod Biol..

[24] Chen C (2012). Classical and nerve-sparing radical hysterectomy: An evaluation of the nerve trauma in cardinal ligament. Gynecol Oncol..

[25] Lee YH (2018). Proteomic Analysis of Pelvic Autonomic Nerve in Nerve-sparing Radical Hysterectomy for Cervical Carcinoma. Cancer Genomics Proteomics..

[26] Zhang X (2017). Intraoperative nerve staining in nerve-sparing radical hysterectomy: a pilot study. Arch Gynecol Obstet..

[27] Katahira A (2005). Intraoperative electrical stimulation of the pelvic splanchnic nerves during nerve-sparing radical hysterectomy. Gynecol Oncol..

[28] Nagai T (2012). Individualized radical hysterectomy procedure using intraoperative electrical stimulation for patients with cervical cancer. Int J Gynecol Cancer..

[29] Chen CL (2010). The measurement of vesical detrusor electromyographic activity during nerve-sparing radical hysterectomy. Reprod Sci..

[30] Li P (2018). The 3D reconstructions of female pelvic autonomic nerves and their related organs based on MRI: a first step towards neuronavigation during nerve-sparing radical hysterectomy. Eur Radiol..

[31] Balaya, V. *et al*. Three-Dimensional Modelization of the Female Human Inferior Hypogastric Plexus: Implications for Nerve-Sparing Radical Hysterectomy. *Gynecol Obstet Invest*. 1–8 (2018). 10.1159/000494255 30380543

[32] Lucidi A (2017). Self-Reported Long-Term Autonomic Function After Laparoscopic Total Mesometrial Resection for Early-Stage Cervical Cancer: A Multicentric Study. Int J Gynecol Cancer..

[33] Magistro G (2017). Emerging Minimally Invasive Treatment Options for Male Lower Urinary Tract Symptoms. Eur Urol..

[34] Glybochko, P. V. *et al*. The role of waterjet dissection in improving erectile function and continence after nerve-sparing prostatectomy. *Urologiia*. 43–49 (2017). 10.18565/urol.2017.1.43-49 28394522

[35] Granick M (2017). Dispersion Risk Associated With Surgical Debridement Devices. Wounds..

[36] Ramirez PT (2018). Minimally Invasive versus Abdominal Radical Hysterectomy for Cervical Cancer. N Engl J Med..

