# Supplementary material for: Identification and injury to the inferior hypogastric plexus in nerve-sparing radical hysterectomy
Source: Sci Rep. 2019 Sep 13;9:13260. doi: 10.1038/s41598-019-49856-w (PMC6744562; doi:10.1038/s41598-019-49856-w)
Supplement: Supplementary file 1 — Supplement Table 1 [file 41598_2019_49856_MOESM1_ESM.docx]

**Article type**

Subgroup analysis from a randomized controlled study

**Title**

Identification and injury to the inferior hypogastric plexus in nerve-sparing radical hysterectomy

**Short title**

Identification and injury to IHP in NSRH

**Authors**

Lei Li, M.D.,^1^ lileigh@163.com

Yalan Bi, M.D.,^2^ biyeye81@126.com

Leiming Wang, M.D.,^3^ wangleiming0918@163.com

Xinxin Mao, M.D.,^2^ pumchmaoxinxin@126.com

Bernhard Kraemer, M.D.,^4^ bernhard.kraemer@med.uni-tuebingen.de

Jinghe Lang, M.D.,^1^ langjh@vip.163.com

Quancai Cui, M.D.,^2^ cuiqc@sina.com

Ming Wu, M.D.,^1^ wuming@pumch.cn

**Dr Lei Li and Dr Yalan Bi contributed equally to the manuscript.**

**Affiliations**

^1^ Department of Obstetrics and Gynecology, Peking Union Medical College Hospital, Peking Union Medical College & Chinese Academy of Medical Science, Beijing 100730, China

^2^ Department of Pathology, Peking Union Medical College Hospital, Peking Union Medical College & Chinese Academy of Medical Science, Beijing 100730, China

^3^ Department of Pathology, Xuanwu Hospital, Capital Medical University, 45# Changchun Street, Beijing 100053, China

^4^ Department of Obstetrics and Gynecology, University of Tuebingen, Calwerstr. 7, Tübingen 72076, Germany

**Corresponding authors**

Ming Wu, M.D.^1^ and Quancai Cui, M.D.^2^

^1^ Department of Obstetrics and Gynecology, Peking Union Medical College Hospital, Peking Union Medical College & Chinese Academy of Medical Science (MW)

^2^ Department of Pathology, Peking Union Medical College Hospital, Peking Union Medical College & Chinese Academy of Medical Science, Beijing 100730, China (QC)

Address: Shuaifuyuan No. 1, Dongcheng District, Beijing 100730, China

Email: wuming@pumch.cn (MW), cuiqc@sina.com (QC)

Phone: 86-139-1198-8831

**Disclosure**

All authors declare that they have no financial or non-financial competing interests to disclose.

Supplement Table 1

Epidemiological and clinical characteristics of the participants

|  | Waterjet group  (*N* =30) | Control group  (*N* =30) | *P* |
| --- | --- | --- | --- |
| Age (year), median (range) | 45 (29-55) | 42 (30-65) | 0.026 |
| Menopause, *N* (%) | 6 (20.0%) | 5 (16.7%) | 0.739 |
| Gravidity, median (range) | 3 (0-6) | 3 (0-6) | 0.583 |
| Parity, median (range) | 1 (0-3) | 1 (0-3) | 0.934 |
| Weight (Kg), mean±SD | 58.9±7.2 | 60.2±8.6 | 0.532 |
| Height (cm), mean±SD | 160.9±5.4 | 162.8±4.4 | 0.134 |
| BMI (Kg/m^2^), mean±SD | 22.7±2.1 | 22.7±3.1 | 0.981 |
| History of pelvic surgeries, *N* (%) | 12 (40.0%) | 13 (43.3%) | 0.793 |
| CA125 (U/ml), median (range) | 14.6 (5.1-1259.8)  (n=9) | 13.4 (8.0-24.8)  (n=10) | 0.414 |
| SCC-Ag (μg/L), median (range) | 1.5 (0.3-10.0)  (n=26) | 0.8 (0.5-7.5)  (n=20) | 0.103 |
| Preoperative chemotherapy | 10 (33.3%) | 7 (23.3%) | 0.390 |
| Cycles, median (range) | 2 (1-2)  (n=10) | 2 (1-3)  (n=7) | 0.270 |
| Preoperative imaging, *N* (%) |  |  |  |
| Involvement of parametrium | 1 (3.3%) | 0 (0.0%) | 0.500 |
| Positive LN | 6 (20.0%) | 7 (23.3%) | 0.754 |
| Conization before RH, *N* (%) | 13 (43.3%) | 8 (26.7%) |  |
| Clinical Stage, *N* (%) |  |  | 0.426 |
| IB1 | 17 (56.7%) | 20 (66.7%) |  |
| IB2 | 13 (43.3%) | 10 (33.3%) |  |

BMI, body mass index. LN, lymph nodes. LVSI, lymph-vascular space invasion. RH, radical hysterectomy. SCC-Ag, squamous cell carcinoma antigen. SD, standard deviation.
