# Supplementary material for: Identification and injury to the inferior hypogastric plexus in nerve-sparing radical hysterectomy
Source: Sci Rep. 2019 Sep 13;9:13260. doi: 10.1038/s41598-019-49856-w (PMC6744562; doi:10.1038/s41598-019-49856-w)
Supplement: Supplementary file 2 — Supplemental Table 2 [file 41598_2019_49856_MOESM2_ESM.docx]

**Article type**

Subgroup analysis from a randomized controlled study

**Title**

Identification and injury to the inferior hypogastric plexus in nerve-sparing radical hysterectomy

**Short title**

Identification and injury to IHP in NSRH

**Authors**

Lei Li, M.D.,^1^ lileigh@163.com

Yalan Bi, M.D.,^2^ biyeye81@126.com

Leiming Wang, M.D.,^3^ wangleiming0918@163.com

Xinxin Mao, M.D.,^2^ pumchmaoxinxin@126.com

Bernhard Kraemer, M.D.,^4^ bernhard.kraemer@med.uni-tuebingen.de

Jinghe Lang, M.D.,^1^ langjh@vip.163.com

Quancai Cui, M.D.,^2^ cuiqc@sina.com

Ming Wu, M.D.,^1^ wuming@pumch.cn

**Dr Lei Li and Dr Yalan Bi contributed equally to the manuscript.**

**Affiliations**

^1^ Department of Obstetrics and Gynecology, Peking Union Medical College Hospital, Peking Union Medical College & Chinese Academy of Medical Science, Beijing 100730, China

^2^ Department of Pathology, Peking Union Medical College Hospital, Peking Union Medical College & Chinese Academy of Medical Science, Beijing 100730, China

^3^ Department of Pathology, Xuanwu Hospital, Capital Medical University, 45# Changchun Street, Beijing 100053, China

^4^ Department of Obstetrics and Gynecology, University of Tuebingen, Calwerstr. 7, Tübingen 72076, Germany

**Corresponding authors**

Ming Wu, M.D.^1^ and Quancai Cui, M.D.^2^

^1^ Department of Obstetrics and Gynecology, Peking Union Medical College Hospital, Peking Union Medical College & Chinese Academy of Medical Science (MW)

^2^ Department of Pathology, Peking Union Medical College Hospital, Peking Union Medical College & Chinese Academy of Medical Science, Beijing 100730, China (QC)

Address: Shuaifuyuan No. 1, Dongcheng District, Beijing 100730, China

Email: wuming@pumch.cn (MW), cuiqc@sina.com (QC)

Phone: 86-139-1198-8831

**Disclosure**

All authors declare that they have no financial or non-financial competing interests to disclose.

Supplement Table 2

Surgical and pathologic characteristics and postoperative adjuvant treatment of the participants

|  | Waterjet group (*N*=30) | Control group (*N* =30) | *P* |
| --- | --- | --- | --- |
| Operation duration (min), median (range) | 200 (130-245) | 180 (130-400) | 0.042 |
| Dissection time of [IHP](javascript:;) (s) |  |  |  |
| Right IHP | 383 (182-594) | 350 (186-540) | 0.240 |
| Left IHP | 397 (186-723) | 348 (195-533) | 0.438 |
| EBS (ml), median (range) | 200 (20-500) | 200 (20-3800) | 0.391 |
| Transfusion (ml), median (range) | 200  (n=1) | 3000  (n=1) | 0.317 |
| Preservation of ovaries, *N* (%) | 12 (40.0%) | 15 (50.0%) | 0.436 |
| Resection extension, median (range) |  |  |  |
| Cardinal ligament (cm) | 3.5±0.7 | 3.6±0.7 | 0.466 |
| Uterosacral ligament (cm) | 3.7±0.6 | 3.7±0.5 | 1.000 |
| Anterior vaginal wall (cm) | 3.8±0.4 | 3.8±0.4 | 0.744 |
| Posterior vaginal wall (cm) | 3.8±0.4 | 3.8±0.4 | 0.759 |
| Hospital stay, median (range) |  |  |  |
| Postoperative stay (days) | 8 (5-14) | 7 (5-32) | 0.255 |
| Total stay (days) | 12 (8-28) | 11 (6-61) | 0.382 |
| Diameter of tumor (mm), median (range) | 40 (10-65) | 25 (10-60) | 0.565 |
| Pathologic subtype, *N* (%) |  |  | 0.604 |
| Squamous carcinoma | 25 (83.3%) | 22 (73.3%) |  |
| Adenocarcinoma | 4 (13.3%) | 7 (23.3%) |  |
| Adenosquamous carcinoma | 1 (3.3%) | 1 (3.3%) |  |
| Residual lesions in RH specimens, *N* (%) | 23 (76.7%) | 25 (83.3%) | 0.519 |
| - Pathological differentiation, *N* (%) |  |  | 0.429 |
| Grade 1 | 5 (16.7%) | 7 (23.3%) |  |
| Grade 2 | 19 (63.3%) | 14 (46.7%) |  |
| Grade 3 | 6 (20.0%) | 9 (30.0%) |  |
| Invasion depth of stroma, *N* (%) |  |  | 0.457 |
| ≤1/3 | 13 (43.3%) | 14 (46.7%) |  |
| 1/3-2/3 | 7 (23.3%) | 10 (33.3%) |  |
| >2/3 | 10 (33.3%) | 6 (20.0%) |  |
| LVSI in RH specimens, *N* (%) | 3 (10.0%) | 6 (20.0%) | 0.472 |
| Involvement of uterine body, *N* (%) | 4 (13.3%) | 3 (10.0%) | 0.500 |
| [Involvement](http://dict.youdao.com/w/eng/parametrial_involvement/#keyfrom=dict.phrase.wordgroup) of parametrium, *N* (%) | 1 (3.3%) | 1 (3.3%) | 0.754 |
| Positive vaginal margin, *N* (%) | 1 (3.3%) | 1 (3.3%) | 0.754 |
| LN metastasis |  |  |  |
| PLN no., median (range) | 26 (10-50) | 31 (9-48) | 0.641 |
| Positive no., *N* (%) | 6 (20.0%) | 3 (10.0%) | 0.472 |
| Common iliac LN no., median (range) | 3 (1-7) | 3 (1-7) | 0.727 |
| Positive no., *N* (%) | 2 (6.7%) | 1 (3.3%) | 0.500 |
| PALN no., median (range) | 13 (6-22) | 13 (6-22) | 0.994 |
| Positive no., *N* (%) | 1 (3.3%) | 1 (3.3%) | 0.754 |
| Postoperative radiotherapy *N* (%) |  |  | 0.370 |
| None | 14 (46.7%) | 12 (40.0%) |  |
| CCRT | 15 (50.0%) | 14 (46.7%) |  |
| Radiotherapy only | 1 (3.3%) | 4 (13.3%) |  |
| Chemotherapy in CCRT, median (range) | 4 (2-6)  (n=15) | 5 (1-6)  (n=14) | 0.288 |
| Postoperative chemotherapy *N* (%) | 1 (3.3%) | 3 (10.0%) | 0.306 |
| Cycles, median (range) | 6  (n=1) | 5 (4-6)  (n=3) | 0.346 |
| Severe complications |  |  |  |
| Perioperative, *N* (%) | 1 (3.3%) | 3 (10.0%) | 0.306 |
| Within 3 months, *N* (%) | 1 (3.3%) | 7 (23.3%) | 0.026 |

CCRT, concurrent chemoradiotherapy. EBS, estimated blood loss. LN, lymph nodes. LVSI, lymph-vascular space invasion. PALN, para-aortic lymph nodes. PLN, pelvic lymph nodes. RH, radical hysterectomy.
