# Supplementary material for: Identification and injury to the inferior hypogastric plexus in nerve-sparing radical hysterectomy
Source: Sci Rep. 2019 Sep 13;9:13260. doi: 10.1038/s41598-019-49856-w (PMC6744562; doi:10.1038/s41598-019-49856-w)
Supplement: Supplementary file 3 — Supplemental Table 3 [file 41598_2019_49856_MOESM3_ESM.docx]

**Article type**

Subgroup analysis from a randomized controlled study

**Title**

Identification and injury to the inferior hypogastric plexus in nerve-sparing radical hysterectomy

**Short title**

Identification and injury to IHP in NSRH

**Authors**

Lei Li, M.D.,^1^ lileigh@163.com

Yalan Bi, M.D.,^2^ biyeye81@126.com

Leiming Wang, M.D.,^3^ wangleiming0918@163.com

Xinxin Mao, M.D.,^2^ pumchmaoxinxin@126.com

Bernhard Kraemer, M.D.,^4^ bernhard.kraemer@med.uni-tuebingen.de

Jinghe Lang, M.D.,^1^ langjh@vip.163.com

Quancai Cui, M.D.,^2^ cuiqc@sina.com

Ming Wu, M.D.,^1^ wuming@pumch.cn

**Dr Lei Li and Dr Yalan Bi contributed equally to the manuscript.**

**Affiliations**

^1^ Department of Obstetrics and Gynecology, Peking Union Medical College Hospital, Peking Union Medical College & Chinese Academy of Medical Science, Beijing 100730, China

^2^ Department of Pathology, Peking Union Medical College Hospital, Peking Union Medical College & Chinese Academy of Medical Science, Beijing 100730, China

^3^ Department of Pathology, Xuanwu Hospital, Capital Medical University, 45# Changchun Street, Beijing 100053, China

^4^ Department of Obstetrics and Gynecology, University of Tuebingen, Calwerstr. 7, Tübingen 72076, Germany

**Corresponding authors**

Ming Wu, M.D.^1^ and Quancai Cui, M.D.^2^

^1^ Department of Obstetrics and Gynecology, Peking Union Medical College Hospital, Peking Union Medical College & Chinese Academy of Medical Science (MW)

^2^ Department of Pathology, Peking Union Medical College Hospital, Peking Union Medical College & Chinese Academy of Medical Science, Beijing 100730, China (QC)

Address: Shuaifuyuan No. 1, Dongcheng District, Beijing 100730, China

Email: wuming@pumch.cn (MW), cuiqc@sina.com (QC)

Phone: 86-139-1198-8831

**Disclosure**

All authors declare that they have no financial or non-financial competing interests to disclose.

Supplement Table 3

Comparison of urodynamic parameters between control group and waterjet group

| Parameters, median (range) | Waterjet group (*N*=30) | Control group (*N*=30) | *P* |
| --- | --- | --- | --- |
| First removal of catheter at 14 days after RH |  |  |  |
| Residual urine volume (ml) | 50 (0-400) | 75 (0-500) | 0.117 |
| Residual urine volume ≤ 100 ml | 25 (83.3%) | 17 (56.7%) | 0.024 |
| Residual urine volume ≤ 50 ml | 20 (66.7%) | 13 (43.3%) | 0.069 |
| Voiding volume (ml) | 200 (50-350) | 200 (50-400) | 0.849 |
| Voiding time (s) | 60 (11-300) | 52 (10-180) | 0.723 |
| Average urinary flow rates (ml/s) | 3.0 (1.0-10.0) | 2.8 (1.7-9.0) | 0.779 |
| Second removal of catheter after RH (days) | 21 (21-28)  (n=5) | 21 (13-42)  (n=13) | 0.334 |
| Pre-operative urodynamics parameters |  |  |  |
| Bladder capacity at the first void sense (ml) | 181 (90-346) | 158 (103-261) | 0.584 |
| Bladder capacity at normal desire to void (ml) | 296 (157-409) | 268 (190-422) | 0.188 |
| Bladder capacity at strong desire to void (ml) | 402 (305-571) | 465 (308-755) | 0.056 |
| Qmax (ml/s) | 25 (11-60) | 28 (11-79) | 0.728 |
| Qave (ml/s) | 14 (4-24) | 14 (7-24) | 0.224 |
| Pves at Qmax (cmH_2_O) | 39 (21-81) | 43 (23-73) | 0.307 |
| Pdet at Qmax (cmH_2_O) | 27 (18-64) | 35 (16-52) | 0.118 |
| Cves at SDV (ml/cmH_2_O) | 60 (23-83) | 49 (24-86) | 0.045 |
| Cdet at SDV (ml/cmH_2_O) | 33 (21-72) | 30 (21-74) | 0.428 |
| Residual urine volume (ml) | 0 (0-0) | 0 (0-0) | 0.078 |
| Postoperative urodynamics parameters |  |  |  |
| Bladder capacity at the first void sense (ml) | 176 (103-301) | 151 (74-274) | 0.110 |
| Bladder capacity at normal desire to void (ml) | 290 (171-445) | 258 (141-454) | 0.284 |
| Bladder capacity at strong desire to void (ml) | 406 (300-577) | 439 (191-647) | 0.371 |
| Qmax (ml/s) | 27 (10-44) | 22 (12-60) | 0.129 |
| Qave (ml/s) | 12 (4-27) | 12 (7-28) | 0.553 |
| Pves at Qmax (cmH_2_O) | 39 (23-80) | 42 (19-72) | 0.491 |
| Pdet at Qmax (cmH_2_O) | 28 (14-67) | 31 (13-54) | 0.318 |
| Cves at SDV (ml/cmH_2_O) | 60 (24-84) | 44 (21-85) | 0.030 |
| Cdet at SDV (ml/cmH_2_O) | 36 (18-69) | 30 (17-70) | 0.160 |
| Residual urine volume (ml) | 0 (0-50) | 0 (0-50) | 0.557 |

Cdet, detrusor compliance. Cves, bladder compliance. Pdet, detrusor pressure. Pves, bladder pressure. Qave, average flow rate. Qmax, maximun flow rate. RH, radical hysterectomy. SDV, strong desire to void.
