# Supplementary material for: Identification and injury to the inferior hypogastric plexus in nerve-sparing radical hysterectomy
Source: Sci Rep. 2019 Sep 13;9:13260. doi: 10.1038/s41598-019-49856-w (PMC6744562; doi:10.1038/s41598-019-49856-w)
Supplement: Supplementary file 4 — Supplement 1 [file 41598_2019_49856_MOESM4_ESM.docx]

**Article type**

Subgroup analysis from a randomized controlled study

**Title**

Identification and injury to the inferior hypogastric plexus in nerve-sparing radical hysterectomy

**Short title**

Identification and injury to IHP in NSRH

**Authors**

Lei Li, M.D.,^1^ lileigh@163.com

Yalan Bi, M.D.,^2^ biyeye81@126.com

Leiming Wang, M.D.,^3^ wangleiming0918@163.com

Xinxin Mao, M.D.,^2^ pumchmaoxinxin@126.com

Bernhard Kraemer, M.D.,^4^ bernhard.kraemer@med.uni-tuebingen.de

Jinghe Lang, M.D.,^1^ langjh@vip.163.com

Quancai Cui, M.D.,^2^ cuiqc@sina.com

Ming Wu, M.D.,^1^ wuming@pumch.cn

**Dr Lei Li and Dr Yalan Bi contributed equally to the manuscript.**

**Affiliations**

^1^ Department of Obstetrics and Gynecology, Peking Union Medical College Hospital, Peking Union Medical College & Chinese Academy of Medical Science, Beijing 100730, China

^2^ Department of Pathology, Peking Union Medical College Hospital, Peking Union Medical College & Chinese Academy of Medical Science, Beijing 100730, China

^3^ Department of Pathology, Xuanwu Hospital, Capital Medical University, 45# Changchun Street, Beijing 100053, China

^4^ Department of Obstetrics and Gynecology, University of Tuebingen, Calwerstr. 7, Tübingen 72076, Germany

**Corresponding authors**

Ming Wu, M.D.^1^ and Quancai Cui, M.D.^2^

^1^ Department of Obstetrics and Gynecology, Peking Union Medical College Hospital, Peking Union Medical College & Chinese Academy of Medical Science (MW)

^2^ Department of Pathology, Peking Union Medical College Hospital, Peking Union Medical College & Chinese Academy of Medical Science, Beijing 100730, China (QC)

Address: Shuaifuyuan No. 1, Dongcheng District, Beijing 100730, China

Email: wuming@pumch.cn (MW), cuiqc@sina.com (QC)

Phone: 86-139-1198-8831

**Disclosure**

All authors declare that they have no financial or non-financial competing interests to disclose.

**Supplement 1**

**1. Neural areas and their proportions among the harvested IHP tissues**

The tissue histology slides subjected to immunohistochemical staining for S100 protein (Beijing Zhongshan Jinqiao Biotechnology Co. Ltd., 23 South Riverside Road, Xicheng, Beijing, China) were scanned at 20X using a Hamamatsu Nanozoomer 2.0 HT scanner (Hamamatsu Photonics K.K., 812, Joko-cho, Higashi-ku, Hamamatsu, Japan). The scanned whole-slide image was first transformed from the YCbCr color space to the RGB color space and then transformed from the RGB color space to the red ratio space as follows: *redratio(i,j)=r(i,j)/(g(i,j)+b(i,j)+s)*, where *r(i,j)*, *g(i,j)* and *b(i,j)* represent the pixel values in the red, green and blue color space, respectively, at the image coordinate *(i,j)*. The red ratio space is a grayscale space to accentuate the S100 staining. In the transformation, *s* is a small constant to avoid dividing by zero. Next, a threshold-based image segmentation method was applied to the red-ratio image to separate the neural tissue regions from other tissue regions. In addition, Gaussian filtering and morphological opening were performed to merge fragmented neural tissue regions. This merging procedure will help the subsequent procedure for the correction of overestimated cross-intersection neural area. Finally, each neural tissue region was extracted, and its area was computed. Small detected regions with areas less than 100 μm^2^ were removed to suppress noise. When multiple neural tissues exist in the same tissue sample, it is difficult to accurately cut each neural tissue along its cross-intersection plane. As a result, some neural tissue regions appear elongated on the slide, and the corresponding neural tissue areas were overestimated. To correct this overestimation, each detected neural tissue region was fit to an ellipse, and any region with a long axis greater than twice the short axis was treated as an elongated neural tissue, whose radius was estimated from its short axis and area was calculated as (*pi*radius^2^*). The total estimated neural tissue area, as well as the total tissue area, was computed for each whole-slide image. The percentage of total area over total tissue was also computed for each whole-slide image.


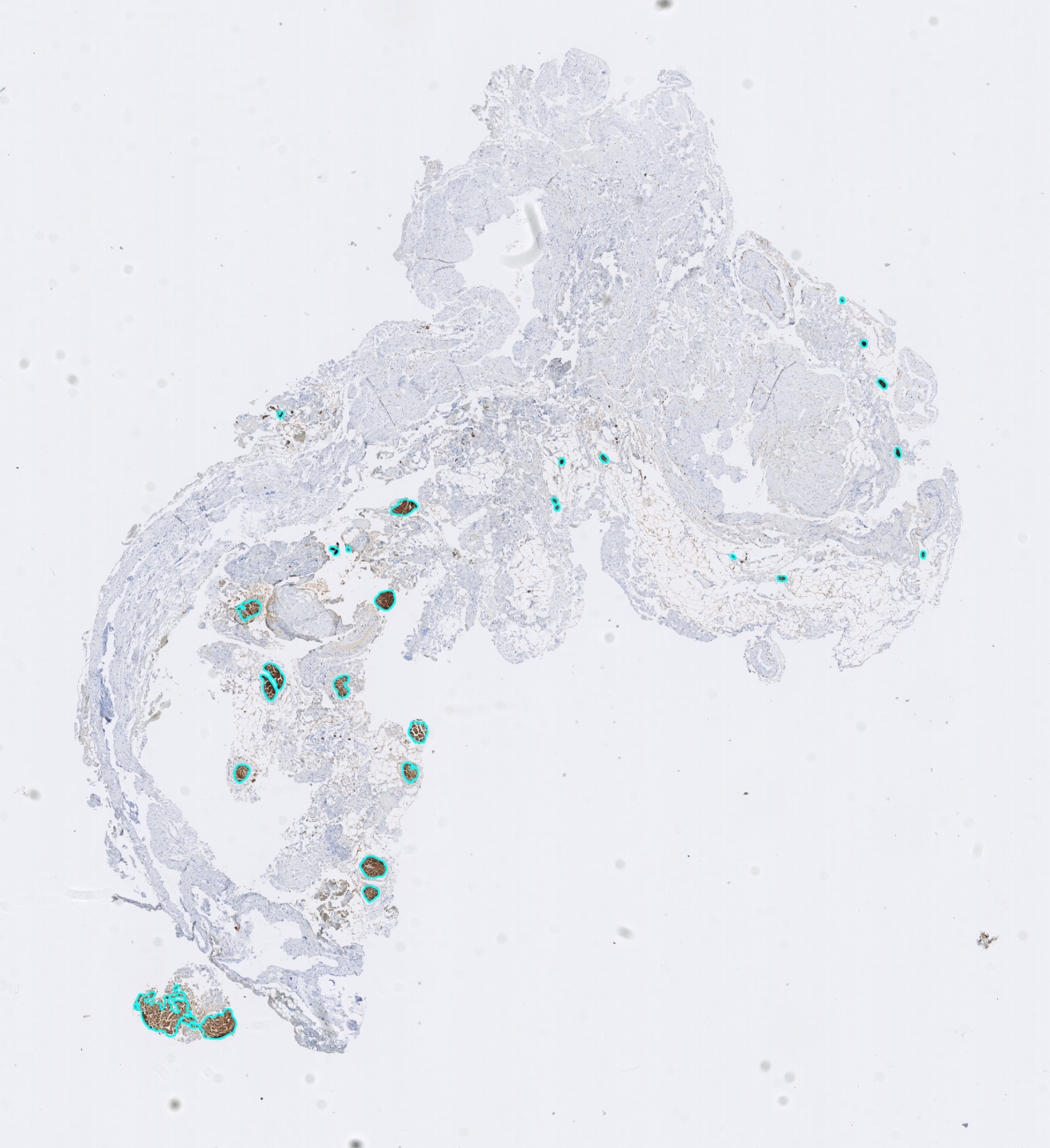


Supplement 1 Figure 1: one example of scanned whole-slide image.


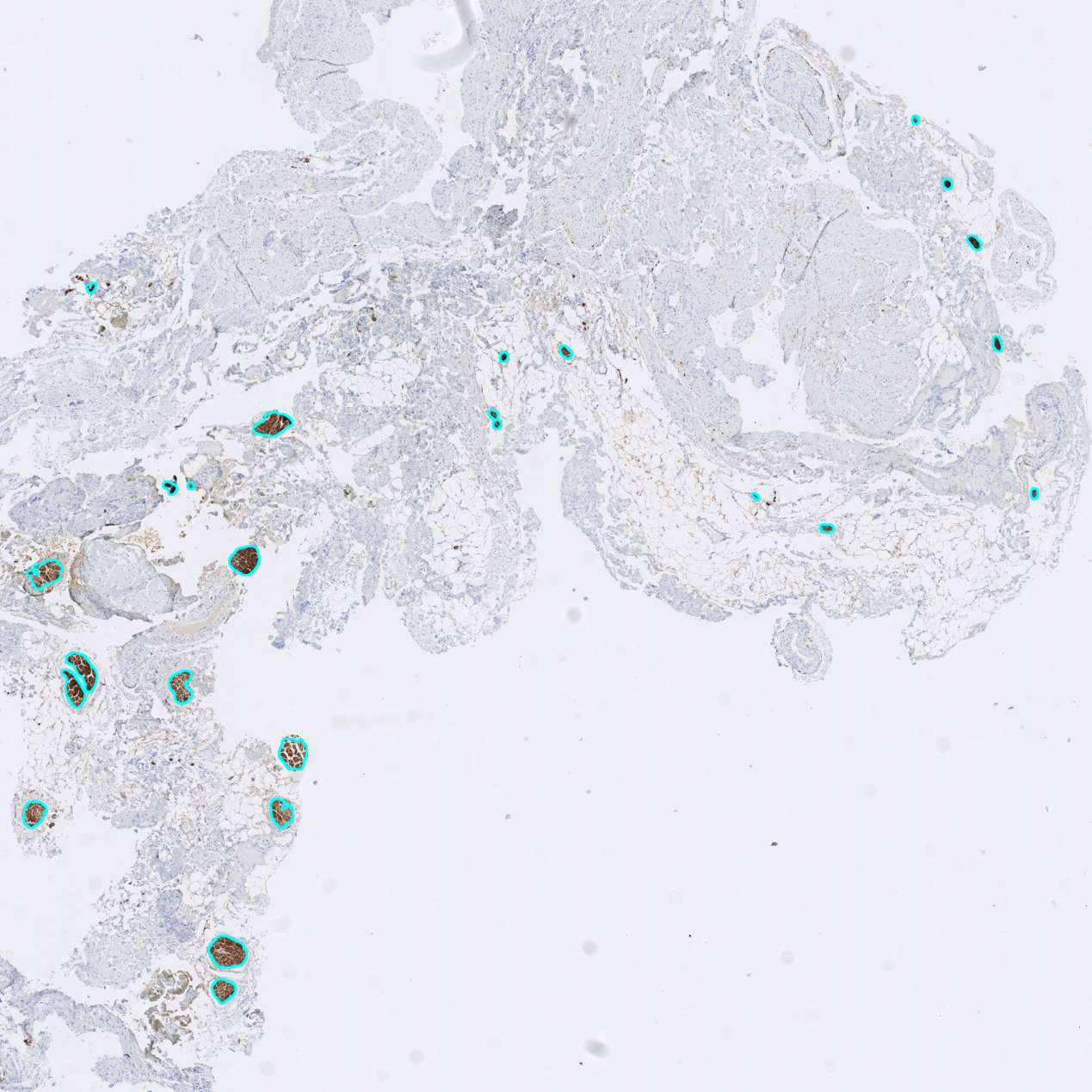


Supplement 1 Figure 2: partial details in Figure 1.

**2. Specific staining of neural tissues**

All tissues were fixed with 3.7% formalin and regularly dehydrated and embedded in paraffin. Slices with a thickness of 4 μm were dewaxed for specific staining.

***(1) Staining of luxol fast blue (LFB)***

After dewaxing, the tissues were dipped in 95% ethyl alcohol and then immersed in fast blue stain in a 60°C water bath for 2-4 hours. Then, the tissues were differentiated by 2% lithium carbonate and stained by hematoxylin eosin staining.


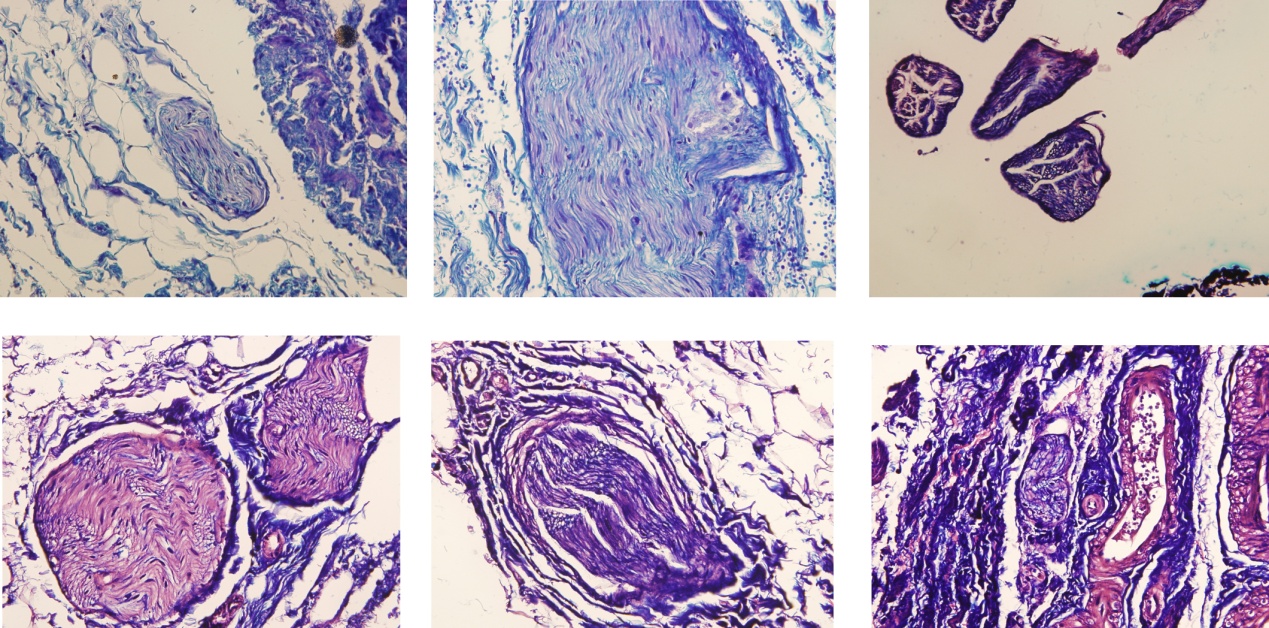


Supplement 1 Figure 3: Different examples of LFB staining. From left to right, up to down, the six figures present the change of normal, normal, mild decreased, mild decreased, moderate decreased and moderate decreased.

***(2) Immunohistochemical staining of myelin basic protein (MBP), neurofilament (NF) and S100 protein***

After dewaxing, the tissues were rehydrated in citrate antigen repair buffer (pH 6.0). Diluted primary antibodies for MBP, NF and S100 were respectively incubated with the tissues overnight for immunohistochemical staining. The ready-made dilution concentration antibodies were from Beijing Zhongshan Jinqiao Biotechnology Co. Ltd. (23 South Riverside Road, Xicheng, Beijing, China).

***(3) Judging the degree of neural tissue impairment***

Approximately one-third loss of the myelin sheath in MBP and LFB staining or one-third loss of neuronal axons on NF staining was defined as a mild decrease. One-third to two-thirds loss and more than two-thirds were defined as moderate and severe decreases, respectively. S100 staining was used to describe the activities of Schwann cells around the peripheral nerves. Mild, moderate and severe increases in S100 staining were defined according to the judgement of the pathologists.


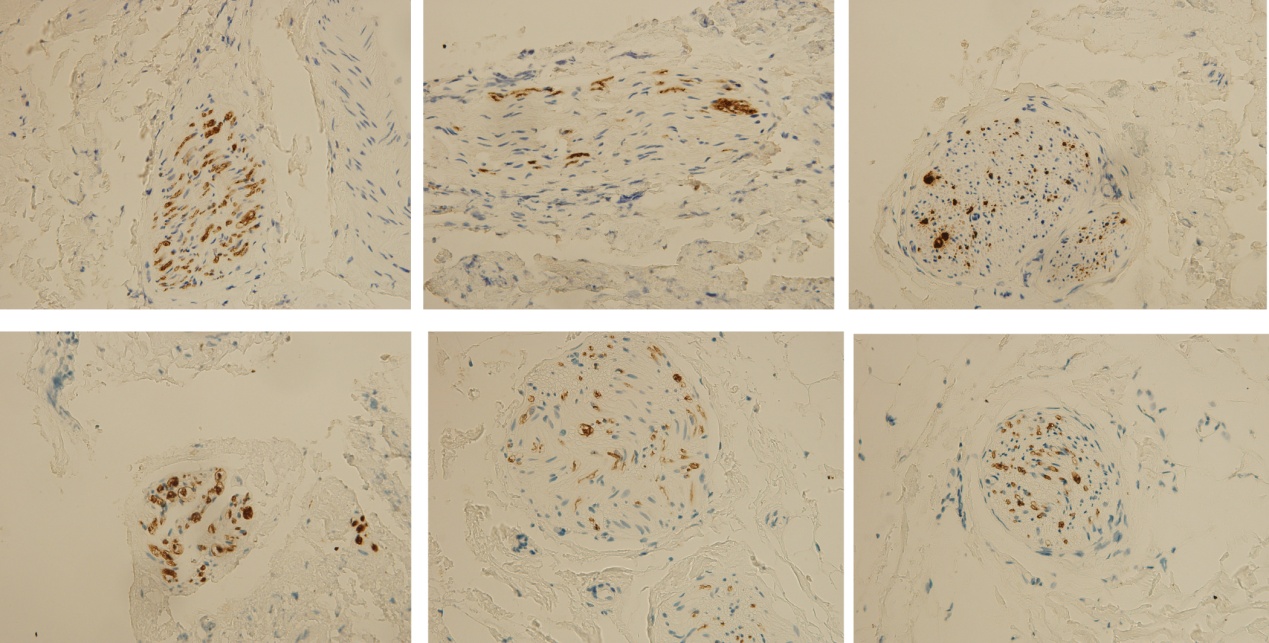


Supplement 1 Figure 4: Different examples of MBP staining. From left to right, up to down, the six figures present the change of normal, mild decreased, mild decreased, normal, moderate decreased and mild decreased.
