# Supplementary material for: Identification and injury to the inferior hypogastric plexus in nerve-sparing radical hysterectomy
Source: Sci Rep. 2019 Sep 13;9:13260. doi: 10.1038/s41598-019-49856-w (PMC6744562; doi:10.1038/s41598-019-49856-w)
Supplement: Supplementary file 7 — Supplement 4 [file 41598_2019_49856_MOESM7_ESM.docx]

**Article type**

Subgroup analysis from a randomized controlled study

**Title**

Identification and injury to the inferior hypogastric plexus in nerve-sparing radical hysterectomy

**Short title**

Identification and injury to IHP in NSRH

**Authors**

Lei Li, M.D.,^1^ lileigh@163.com

Yalan Bi, M.D.,^2^ biyeye81@126.com

Leiming Wang, M.D.,^3^ wangleiming0918@163.com

Xinxin Mao, M.D.,^2^ pumchmaoxinxin@126.com

Bernhard Kraemer, M.D.,^4^ bernhard.kraemer@med.uni-tuebingen.de

Jinghe Lang, M.D.,^1^ langjh@vip.163.com

Quancai Cui, M.D.,^2^ cuiqc@sina.com

Ming Wu, M.D.,^1^ wuming@pumch.cn

**Dr Lei Li and Dr Yalan Bi contributed equally to the manuscript.**

**Affiliations**

^1^ Department of Obstetrics and Gynecology, Peking Union Medical College Hospital, Peking Union Medical College & Chinese Academy of Medical Science, Beijing 100730, China

^2^ Department of Pathology, Peking Union Medical College Hospital, Peking Union Medical College & Chinese Academy of Medical Science, Beijing 100730, China

^3^ Department of Pathology, Xuanwu Hospital, Capital Medical University, 45# Changchun Street, Beijing 100053, China

^4^ Department of Obstetrics and Gynecology, University of Tuebingen, Calwerstr. 7, Tübingen 72076, Germany

**Corresponding authors**

Ming Wu, M.D.^1^ and Quancai Cui, M.D.^2^

^1^ Department of Obstetrics and Gynecology, Peking Union Medical College Hospital, Peking Union Medical College & Chinese Academy of Medical Science (MW)

^2^ Department of Pathology, Peking Union Medical College Hospital, Peking Union Medical College & Chinese Academy of Medical Science, Beijing 100730, China (QC)

Address: Shuaifuyuan No. 1, Dongcheng District, Beijing 100730, China

Email: wuming@pumch.cn (MW), cuiqc@sina.com (QC)

Phone: 86-139-1198-8831

**Disclosure**

All authors declare that they have no financial or non-financial competing interests to disclose.

**Figure legends**

Figure 1

Flow diagram of the study. IHP, inferior hypogastric plexus. NSRH, nerve-sparing radical hysterectomy.

Figure 2

Illustration of the right parametrium. (A) The drainage of the inferior vesical vein (IVV) and middle vesical vein (MVV) to the deep uterine vein (DUV), an anatomic landmark of the cardinal ligament, is displayed. (B) After removal of the vessels, the inferior hypogastric plexus (IHP) with its vesical and uterine branches were displayed. In our study, the uterine branches of the IHP were resected for pathological evaluation, and the vesical branches were deliberately reserved.

Figure 3

An illustration of quantitative analysis of the neural tissue proportion in a cross section of uterine branches of the inferior hypogastric plexus (×20). The indigo circles denote the neural tissue captured by the image segmentation method. A more detailed description is provided in Supplement 1.

Figure 4

Images of specific staining for nerve tissues. (A) Normal myelin in luxol fast blue (LFB) staining. (B) Mild myelin decrease in LFB staining. (C) Normal expression of myelin basic protein (MBP). (D) Mild myelin decrease in MBP expression. (E) Normal expression of neurofilament (NF). (F) Mild decrease of NF expression.

**Table legends**

Table 1

Quantitative analysis and staining of uterine branches of the inferior hypogastric plexus. Every patient had two samples (left and right) for analysis and comparison. For more detailed data, refer to Supplemental Table 5. NF, neurofilament. MBP, myelin basic protein. LFB, luxol fast blue.

Supplemental Table 1

Epidemiological and clinical characteristics of the participants

Supplemental Table 2

Surgical and pathological characteristics and postoperative adjuvant treatment of the participants

Supplemental Table 3

Comparison of urodynamic parameters between the control and waterjet groups

**Legends for supplement materials**

Supplement 1

Detailed methods used for calculation of neural areas and proportion and description of impairment degrees of the neural tissues

Supplement 2

Raw data of the participants.

Supplement 3

Raw data of the sampling, quantitative analysis, and specific staining of the uterine branches of the inferior hypogastric plexus.

Supplement 4

Legends for figures, table, supplement tables and supplement materials.
